# Real-Time Optical Monitoring of Endotracheal Tube Displacement

**DOI:** 10.3390/bios10110174

**Published:** 2020-11-12

**Authors:** Ramzan Ullah, Karl Doerfer, Pawjai Khampang, Faraneh Fathi, Wenzhou Hong, Joseph E. Kerschner, Bing Yu

**Affiliations:** 1 Department of Biomedical Engineering, Marquette University and Medical College of Wisconsin, Milwaukee, WI 53233, USA; ramzan.ullah@marquette.edu (R.U.); faraneh.fathi@marquette.edu (F.F.); 2 Departments of Microbiology and Otolaryngology, Medical College of Wisconsin, Milwaukee, WI 53226, USA; kdoerfer@mcw.edu (K.D.); pkhampang@mcw.edu (P.K.); whong@mcw.edu (W.H.); JKerschner@mcw.edu (J.E.K.)

**Keywords:** endotracheal tube, displacement monitoring, fiber optic sensor

## Abstract

Proper ventilation of a patient with an endotracheal tube (ETT) requires proper placement of the ETT. We present a sensitive, noninvasive, operator-free, and cost-effective optical sensor, called Opt-ETT, for the real-time assessment of ETT placement and alerting of the clinical care team should the ETT become displaced. The Opt-ETT uses a side-firing optical fiber, a near-infrared light-emitting diode, two photodetectors with an integrated amplifier, an Arduino board, and a computer loaded with a custom LabVIEW program to monitor the position of the endotracheal tube inside the windpipe. The Opt-ETT generates a visual and audible warning if the tube moves over a distance set by the operator. Displacement prediction is made using a second-order polynomial fit to the voltages measured from each detector. The system is tested on ex vivo porcine tissues, and the accuracy is determined to be better than 1.0 mm. In vivo experiments with a pig are conducted to test the performance and usability of the system.

## 1. Introduction

An endotracheal tube (ETT) is a pliable plastic duct placed into a patient’s trachea to facilitate mechanical ventilation when a person is unable to breath on their own. This occurs in situations such as when anesthesia or substantial sedation is administered during surgery, when a patient is in the intensive care unit, or when a patient suffers from some other injury or pathologic process [1,2]. Correct positioning of the ETT inside the trachea to fully ventilate the lungs is critical [3,4,5]. Improper placement of the ETT may cause severe complications or even death, particularly in newborns and children. When an ETT does become displaced, prompt intervention is required to correct the position, and depending upon the degree of displacement, replacement of the ETT may be quite difficult and associated with patient complications [6,7,8]. A simple and cost-effective technology to rapidly alert clinical care teams of ETT displacement, particularly minor displacements that may signal a more significant displacement (extubation), does not currently exist. There are several techniques for clinical confirmation of the ETT position. Chest X-ray (CXR) is the current gold standard, but it has several drawbacks, including repeated exposure to radiation, the need for specialized equipment that generally requires interruption of patient care, and the ability to provide ETT position information only at a single time point [3,9,10]. In patients who are intubated, respiratory function (tidal volume, airway pressure, and flow), peripheral oxygen levels, and ETT carbon dioxide levels are generally measured as a proxy for appropriate placement of an ETT and proper ventilation. However, these measures do not provide specific or immediate feedback on ETT placement and can provide information only very late in a serious difficulty related to the ETT position [11,12,13]. Other methods for determining ETT placement have been proposed, such as ultrasound or fiberoptic visualization; however, these techniques are quite limited in actual clinical practice.

There are different methods to secure an ETT after placement [14,15,16], but keeping the tube at the correct position in various situations, such as those in which a patient must be moved or transported, may be challenging [7,8,17,18,19,20,21,22,23,24]. There are specific patient circumstances such as surgical reconstruction of the airway or trachea or a limited distance for proper placement, especially in children and neonates, which increase the critical nature of maintaining the proper placement of an ETT. Displacement of the ETT can result in distal movement of the tube, which may result in mainstem bronchi ventilation and primary aeration of only one lung. Often, identifying this difficulty happens later in a patient’s clinical course and can be associated with significant complications. In its worst-case scenario, ETT displacement can lead to extubation of the patient, which, even if identified quickly, can lead to serious complications and death [25,26]. In each of these types of situations, a monitoring system to easily and continuously confirm ETT position would provide an important adjunct to clinical care and would have the ability to improve patient outcomes and limit patient complications. Therefore, there is an unmet clinical need for an ETT monitoring system that can issue an alert to the care team if the tube is displaced or dislodged beyond a desired distance. This system must be able to predict the estimated displacement from the initial point so that the care team can correct the position quickly before complications occur.

Here, we report an optical system, Opt-ETT, for the real-time monitoring of ETT placement and to provide immediate feedback in the event of ETT displacement. The Opt-ETT consists of a side-firing optical fiber attached to the ETT for trachea illumination and two photodiodes with an integrated operational amplifier placed on the chest skin above the tube for light detection. Tissue is an optically turbid medium in which light waves attenuate exponentially with the distance traveled. As the tube moves, photodiodes detect changes in the intensity of light transmission through the tracheal tissue, and a LabVIEW program generates an alarm if the displacement exceeds a preset value. Ex vivo porcine tissues are used to characterize the device for both longitudinal and rotational movements, and the results are compared with those of the simulated signal. In vivo experiments are also conducted in pigs to test the usability and intersubject variability of the device.

## 2. Materials and Methods

### 2.1. Optical Monitoring System

**Figure 1:**
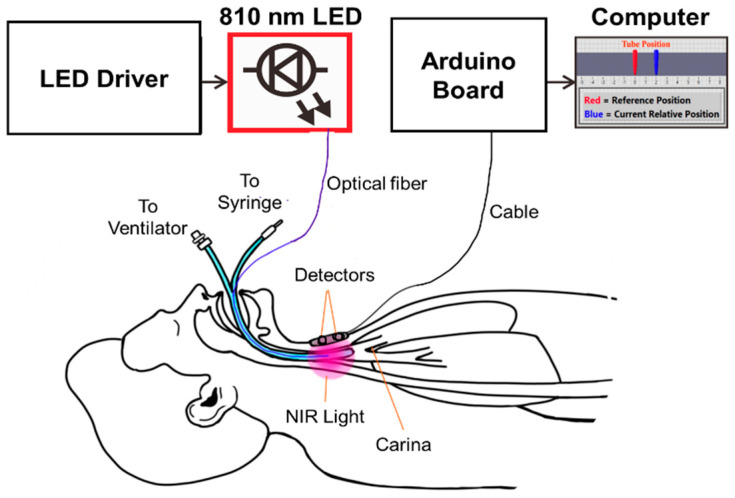
Principle schematics of the optical sensor (Opt-ETT) system and the intended use for endotracheal tube (ETT) monitoring.

The operating principle of the Opt-ETT system and the intended use are illustrated in Figure 1. The Opt-ETT includes three parts: the illumination mechanism, the detection circuitry, and data acquisition and processing. The illumination mechanism consists of a 200 µm side-firing optical fiber attached to the ETT and an 810 nm fiber-coupled LED (THORLABS, M810F2) with a power-adjustable driver. The power out of the 200 µm fiber is in the range of 0~2.3 mW. This power is about two-orders lower than the maximum permissible exposure (0.2–0.4 W/cm^2^) of near-infrared light for skin by the IEC60825 and ANSI Z136.1 standards. The 810 nm LED was selected for its considerable penetration depth and to minimize the effects of blood oxygenation because it is near the isosbestic wavelength of hemoglobin. The size of the ETT used in this study is 7 mm without a cuff. The detection system consists of two photodiodes (~8 mm apart) with an integrated transimpedance amplifier (UDT-455, OSI-Optoelectronics, Hawthorne, CA). The photodiodes have a responsivity of ℜ = 0.6 A/W at 810 nm, a spectral range of 350–1100 nm, and a view angle of 50 degrees. A 3 MΩ resistor (*R*) and a 0.01 µF capacitor are added to the feedback network of each amplifier. The output voltages from the detectors, V_1_ and V_2_, can be calculated as V_1,2_ = P_1,2_ × ℜ × R, where P_1,2_ is the light intensity seen by each sensor. The cutoff frequency of this circuit is 5.3 Hz, which is inherently good for the setup because the ETT will unlikely move at a higher frequency. The detector board is confined in a 3D-printed dark enclosure, which is attached to the chest skin above the trachea, where the maximum signal is achieved for detector 2 (i.e., detector 2 is aligned with the tip of the side-firing fiber).

An Arduino board (MEGA2560) is used for data acquisition, and a LabVIEW program is developed to process the signals and display the displacement information. The analog inputs of MEGA2560 have 10-bit resolution with a detection range of 0.005–5.0 V. An essential aspect of the LabVIEW program is the interface, where the user can enter preset displacement values corresponding to V1 and V2. A 2nd-order polynomial fit is then performed, and the system displays the position of the tube in real time. If the tube is moved beyond the preset value, it will set the alarm off.

### 2.2. Simulation of Light Sensitivity to Displacement

To understand the light transmission through porcine tissue and the theoretical sensitivity to displacement, simulations have been performed using a multilayer Monte Carlo model developed by Scott Prahl and Steve Jacques [27]. The tissue model included a layer of adipose-rich tissue and a second layer of porcine muscle-rich tissue. The absorption coefficients (µ_a_) and reduced scattering coefficients (µ_s_’) of the ex vivo porcine tissue at 810 nm were independently measured using a frequency-domain near-infrared spectroscopy (FD-NIRS) instrument [28]. The tissue geometry and optical properties are presented in Table 1. A uniform beam of ~20 million photons from an optical fiber was launched into the tissue at a specific source position (x = 0, y = 0, z = 0). The normalized transmission was plotted against the x position at z = 1.8 cm (or on the side opposite the tissue).

### 2.3. System Characterization of Ex Vivo Porcine Tissues

**Figure 2:**
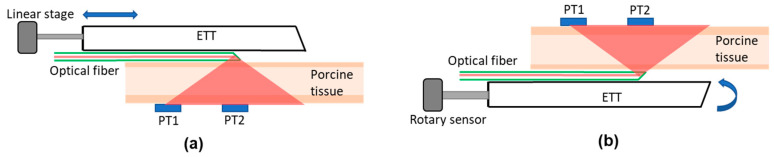
Experimental setups for the ex vivo studies with porcine tissue: (**a**) longitudinal tube displacement and (**b**) tube rotation.

Ex vivo porcine tissues were used to characterize the accuracy and dynamic range of the Opt-ETT system and to compare the measured and simulated responses. The tissues included mixed adipose and muscle tissue of 1.8 cm in thickness. Figure 2 illustrates the experimental setups for the ex vivo studies. For the longitudinal displacement measurement (Figure 2a), a translational displacement sensor (SP1-50, TE connectivity, Chatsworth, CA, USA) was attached to the proximal end of the ETT. The detection box was placed on the bench facing up. The porcine tissue was placed on top of the detection box, and the ETT with the side-firing optical fiber was translated over it with the LED light beam going down. This setup mimics a tube moving longitudinally inside a trachea. To ensure that the tube runs over the porcine tissue in a straight line, the tube was fixed on a linear translation stage that was restricted to move in one dimension only. The setup was modified later to imitate the rotation of an ETT inside a trachea (Figure 2b), in which tube rotation was detected by a decrease in the voltages of the detectors from their peak intensity (or zero rotation). The actual angle of rotation was confirmed by using a rotational sensor (640GS103B06NBAY, Honeywell, Charlotte, NC) attached to the proximal end of the tube. Because the ETT was curved, a metal bar was inserted into the tube to keep it straight. The proximal end of the tube was also restricted so that it could only be rotated. The tube was placed on a black surface with the side-firing fiber facing up, and the pork tissue was placed on it. The detection box was placed on top of the tissue. The tube was rotated over a complete 360° angle, and the outputs of the detectors were recorded.

### 2.4. In Vivo Experiments

**Figure 3:**
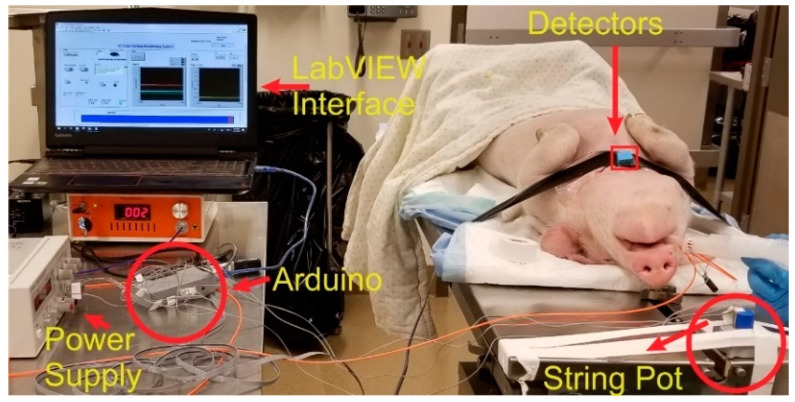
Photograph of the in vivo experiment with the Opt-ETT tube placed in the trachea of a pig and the detector box attached to the chest above the fiber tip.

An in vivo study was conducted on a pig weighing 20.8 kg to test the performance and usability of the Opt-ETT system for linear and rotation displacement detection. The study was approved by the Medical College of Wisconsin (MCW) Institutional Animal Care and Use Committee (IACUC). The MCW Biomedical Resource Center (BRC) veterinary staff performed the anesthetic injection, intubation, and monitoring procedures. Figure 3 is a photo of the experimental setup for the pig study. An ETT with an attached side-firing optical fiber was inserted into the pig’s trachea and tested for the right position using the ventilator. The detection box was placed on the chest with the two detectors along the tube and detector 2 directly above the fiber tip (i.e., maximum signal for V2). The detection box was covered with dark tape to block the room light. The commercial displacement sensor was attached to the proximal end of the tube. The tube was then pushed in and pulled out to record the output voltages (V1 and V2) versus displacement. Special care was taken not to rotate the tube during the linear displacement measurements. V1 and V2 and their corresponding displacement values were saved for a 2nd-order polynomial fit. To measure the voltages against the rotation of the ETT, the tube was marked at different angles. The ETT was then rotated inside the trachea manually, and corresponding V1 and V2 readings were saved at each angle. The tube was rotated with utmost care to minimize any longitudinal movement.

## 3. Results

### 3.1. Simulation Results

**Figure 4:**
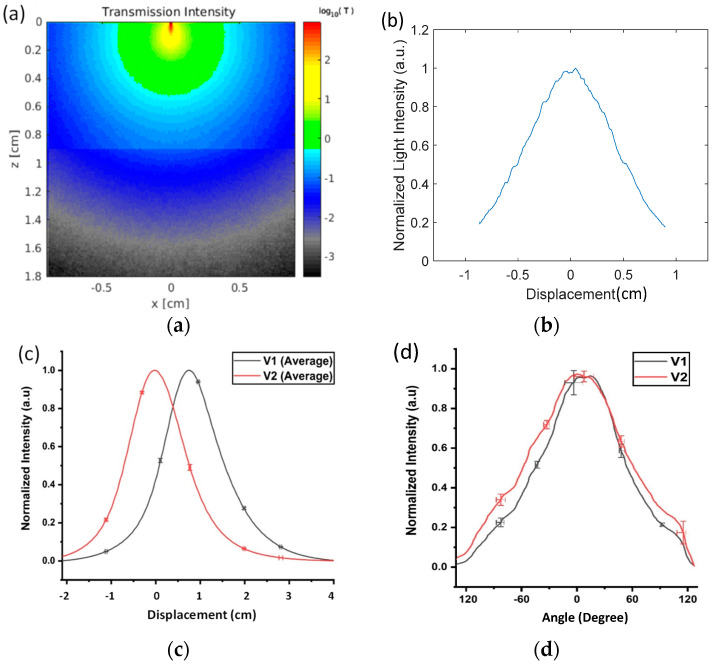
Simulation and ex vivo experimental results from adipose- and muscle-rich porcine tissues: (**a**) simulated photon distribution inside the tissue, (**b**) simulated light transmission vs. displacement, and normalized average voltages (V1 and V2) (**c**) from 16 repeated measurements in response to the translation of the optical fiber against the porcine tissue and (**d**) from 10 repeated measurements in response to the fiber rotation against the tissue.

The simulated results of light transmission through adipose and muscle porcine tissues when the ETT was linearly displaced are shown in Figure 4a,b. The intensity of the light transmission was highest when the detector was aligned with the tip of the optical fiber, which occurred at a displacement of 0 mm. The intensity decreased exponentially with the displacement from the peak and reached 20% at x = ±9 mm.

### 3.2. Ex Vivo Experiments

A total of 16 repeated measurements were taken from the ex vivo porcine tissue at a displacement from −20 to +40 mm. The outputs were normalized by the voltage at the peak for each detector to achieve a standard reference scale across different measurements. The average voltages and standard deviations (STDs) of the 16 measurements for each sensor are plotted in Figure 4c. The maximum STDs were 0.08 V for V1 and 0.14 V for V2. The relevant STDs in V1 and V2 were used to calculate the respective errors in the displacement measurements. A few selected error bars are included in Figure 4c as a reference. The maximum displacement errors were determined to be 0.21 mm for detector 1 and 0.41 mm for detector 2. As the reference point, the peak value of V2 was set for 0 displacement. V2 dropped to 50% when the ETT moved 7 mm to the left from the peak. V1 and V2 dropped down to near 0 beyond a 20 mm displacement on the left. When the ETT moved from the 0 position to the right, V2 continuously decreased, while V1 started at approximately 50% intensity, peaked at a 7 mm displacement, and fell back to 50% intensity at a displacement of 15 mm. Both V1 and V2 fell below the lowest detectable voltage above a 40 mm displacement.

Setting the normalized cutoff intensity at 0.05, the detectable displacement for V2 alone ranged from −17 to +20 mm, covering a total displacement of 37 mm. Similarly, V1 could detect displacements from −11 to +28 mm, covering a total displacement of 39 mm. When both V1 and V2 were used (i.e., V2/V1) to determine the direction of the move, the effective displacement range was reduced to 31 mm (or −11 to +20 mm). Both V1 and V2 followed the general trend of the Monte Carlo simulations in Figure 4b—i.e., the output voltage curves presented a high value when the sensor was aligned with the tip of the illumination fiber and fell off quickly when the tube was moved away from the line of sight of the illumination. V1 and V2 decreased slightly more slowly than the simulated decrease in Figure 4b. This discrepancy is likely due to the errors in measuring the tissue optical properties and due to the detectors having a much larger active area than the bin size used in the simulations. A second-order polynomial fit can be used to predict the displacement from the given voltages from the two sensors.

The normalized mean voltages and STDs measured from the two detectors with the rotation in the range of −120°–+120° are shown in Figure 4d. The maximum STD out of a total of 10 repeated measurements was 0.061 in V1 and 0.057 in V2. The respective errors in displacement at the peaks of the curves were calculated to be 9.7° for V1 and 10.4° for V2. Figure 4d indicates that approximately 50% of the intensity of V1 dropped over a rotation of 54° towards the right and 45° to the left side of the peak value. Similarly, the V2 intensity decreased to 50% of the peak value at 61° on the right and 58° on the left. Using a cutoff voltage of 0.05, the maximal detectable rotation range was determined to be −115–119° for V1 and −135–123° for V2.

### 3.3. In Vivo Pig Experiments

**Figure 5:**
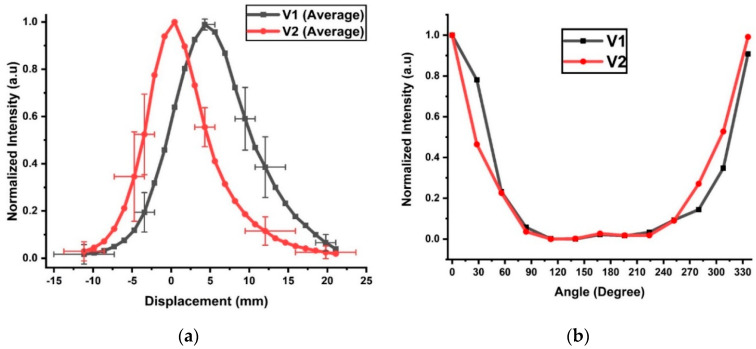
Normalized voltages measured by the two detectors V1 and V2 against the displacement (**a**) and rotation (**b**) of the ETT with a side-firing optic fiber.

The normalized average voltages V1 and V2 against displacement from the in vivo pig study are shown in Figure 5a. The maximum STDs are 0.40 for V1 and 0.54 for V2. The selected horizontal error bars include the maximum displacement uncertainty of 3.9 mm for both V1 and V2, but they occurred at different tube positions. The maximum uncertainty in displacement occurred at −10 mm for V1 but at 20 mm for V2. The high uncertainty at these positions was likely due to the low signal-to-noise ratio (SNR). The shapes of the voltage curves were Gaussian in nature, and high uncertainty in the voltage did not necessarily produce high uncertainty in the displacement. For example, the highest voltage uncertainty occurred at a displacement of −5 mm for V1 and 12 mm for V2, but their respective displacement errors were lower than those in the low SNR regions with normalized signals below 0.1. For V2, a 50% intensity decrease from its peak value occurred at 3 mm on the left and 4 mm on the right. The V1 peak value occurred at 4 mm and decreased by 50% at 0 mm and 10 mm displacements. At a normalized intensity of 0.05, V2 indicated a displacement of 11 mm on the left or 16 mm on the right, spanning a total distance of 27 mm. The cutoff for V1 occurred at −8 and +20 mm, covering a full displacement range of 28 mm. The covered displacement ranges of V1 and V2 at the cutoff intensity were significantly lower than the respective ranges of V1 (41 mm) and V2 (38 mm) observed in the ex vivo tissues (Figure 4c). This difference can be attributed to the higher hemoglobin concentrations in the live tissues and an extra layer of skin, which was not included in the ex vivo study. When both detectors were used for direction detection, the detection range was reduced to −8–+16 mm, or an effective range of 24 mm. The uneven measurements on the two sides of the peaks were likely caused by varying thicknesses of the tissue from the left and right.

The use of the rotational sensor in the in vivo experiment was more challenging because no translation could be involved. Therefore, the ETT was manually turned at increments of 30°. The normalized intensities from V1 and V2 are shown in Figure 5b. The peak intensities were set at 0° for both V1 and V2. The intensities fell sharply near 0°/330° but remained low between 80° and 250°. The signal was detectable up to an angular displacement of ±80° from 0° along the tube. In contrast, the detectable rotation range for the ex vivo tissue was −115–119° for V1 and −123°–135° for V2. The difference might have occurred because the ex vivo tissue was flat, while the trachea was cylindrical. The cylindrical shape of the trachea and friction might have distorted the ETT, resulting in some measurement errors in the angular displacement.

## 4. Discussion

Incorrect positioning, displacement or dislodgement of the ETT may cause insufficient oxygen delivery to the subject and may result in permanent damage to vital organs if it goes unnoticed even for a short period of time. It is commonly accepted that accurate positioning of the ETT in the trachea should be confirmed immediately after placement, and various secondary measures of ventilation are currently utilized to inform clinical care teams of ongoing accurate placement of the ETT. Unfortunately, these measures can provide either incomplete or delayed information such that movement of an ETT results in patient complications. The Opt-ETT is based on the diffusive transmission of NIR light from the ETT through the trachea and to the patient’s chest wall, which attenuates exponentially with the distance from the light source and thus is very sensitive to movement of the tube (or light source) in the trachea. The device can easily achieve millimeter or even submillimeter accuracy. Because a warning signal can be generated if the tube moves over a preset value, the Opt-ETT does not require additional clinical personnel to assess or monitor this output and information to be sent to the clinical team. An alarm signal can simply be sent to a care team leader if there is any appreciable change in the tube position. The Opt-ETT uses a simple LED, a photodetector, an optical fiber, and an Arduino board and thus can be made very simple to use and cost-effective for continuous monitoring in operating rooms, intensive care units, and other clinical settings.

To our knowledge, the Opt-ETT is the first of its kind that aims to continuously and noninvasively monitor ETT displacement and rotation so that care teams are immediately notified if the tube moves beyond a preset limit to allow for rapid correction. The Opt-ETT uses NIR light energy for sensing, which has large penetration capability in biological tissue, operates at very low power, and is safe to both patients and clinical personnel. The side-firing optical fiber embedded in the ETT wall for light delivery neither obstructs air flow inside the tube nor increases the size of the tube diameter. The detection photodetectors allow detection of the fiber tip (thus the tube tip) and rotation in the simplest and most cost-effective way. Finally, embedding the optical fiber into the ETT wall and the simple design of the detector board makes it easy for mass production, further reducing the cost for an ETT procedure.

There are limitations to the current study. While there is no theoretical limit on the minimum detectable displacement of the Opt-ETT, the actual limit is imposed by the low resolution (10 bits) of the data acquisition in the Arduino board. The commercial displacement sensor used for calibration in the ex vivo and in vivo studies was designed for a maximum displacement of 150 cm at very low resolution. The resolution of the Opt-ETT can be improved by using a higher-resolution data acquisition card and a calibration sensor with a smaller range. The maximum output power of the 810 nm LED in the present system is only 2.3 mW. The light throughput can be significantly reduced in larger trachea and thicker tissue in adult patients, which can lead to a signal below the noise floor. The throughput can be improved by increasing the gain of the amplifier or using a higher-power LED or laser diode connected to the side-firing fiber as the source. It is also possible to design a variable gain transimpedance amplifier for different patient ages or body sizes. Another limitation of the system is the relatively small effective detection range (24 mm) for the in vivo tissue, beyond which the signal in one of the two detectors is lost. This small detection range can be increased by using more detectors along the ETT, a higher power source, and better data acquisition. Finally, the size of the detector box is 2.5 × 2 × 1 cm^3^, which is not patient friendly and makes it difficult to maintain good contact with the skin. A flexible, sticker-like sensor would significantly improve the clinical usability of the device and thus will be investigated in our future studies.

## 5. Conclusions

There is an unmet clinical need for a continuous assessment of the ETT position that is prompt, safe, and easy to use. We developed an Opt-ETT device that consists of a side-firing fiber glued onto the tube for illumination; photodiodes with an integrated operational amplifier in transimpedance mode for light transmission detection; and an Arduino board and a LabVIEW interface for data acquisition, analysis, and display. The system was tested on both ex vivo and in vivo porcine tissues. The ex vivo response of the device followed the general trend of the simulated light intensity. The in vivo test demonstrated the feasibility of measuring both linear displacement and rotation with reasonable accuracy. The device is disposable, can be mass-produced, is cost-effective, and thus can be used in resource-poor settings.

## Figures and Tables

**Table 1:** The geometry and optical properties of the ex vivo tissue used in the Monte Carlo simulation.

Tissue Type	Absorption Coeff (cm^−1^)	Scattering Coeff (cm^−1^)	Anisotropy Factor g	Thickness (cm)
Adipose-rich	0.22	25	0.79	0.9
Muscle-rich	0.35	8.3	0.90	0.9

## References

[1] Haas C.F., Eakin R.M., Konkle M.A., Blank R. (2014). Endotracheal Tubes: Old and NewDiscussion. Respir. Care.

[2] Ahmed R.A., Boyer T.J. (2020). Endotracheal Tube (ET). StatPearls.

[3] Rudraraju P., Eisen L.A. (2009). Analytic review: Confirmation of endotracheal tube position: A narrative review. J. Intensive Care Med..

[4] Pollard R.J., Lobato E.B. (1995). Endotracheal tube location verified reliably by cuff palpation. Anesth. Analg..

[5] Kollef M., Legare E.J., Damiano M. (1994). Endotracheal tube misplacement: Incidence, risk factors, and impact of a quality improvement program. South. Med. J..

[6] Kapadia F.N., Tekawade P.C., Nath S.S., Pachpute S.S., Saverkar S.S., Bhise R.A., Chavan A.C., Varghese S.J., Kantak V.U., Kshirsagar R.V. (2014). A prolonged observational study of tracheal tube displacements: Benchmarking an incidence <0.5–1% in a medical-surgical adult intensive care unit. Indian J. Crit. Care Med. Peer Rev. Off. Publ. Indian Soc. Crit. Care Med..

[7] Rajendram R., Khan M.F., Joseph A. (2017). Tracheostomy tube displacement: An update on emergency airway management. Indian J. Respir. Care.

[8] Hyzy R.C. (2017). Complicantions of the endotracheal tube following initial placament: Prevention and management in adult intensive care unit patients. Up Date Pulm. Crit. Care Med..

[9] Sharma D., Tabatabaii S.A., Farahbakhsh N. (2019). Role of ultrasound in confirmation of endotracheal tube in neonates: A review. J. Matern. Fetal Neonatal Med..

[10] Hiles M., Culpan A.-M., Watts C., Munyombwe T., Wolstenhulme S. (2017). Neonatal respiratory distress syndrome: Chest X-ray or lung ultrasound? A systematic review. Ultrasound.

[11] Grmec Š. (2002). Comparison of three different methods to confirm tracheal tube placement in emergency intubation. Intensive Care Med..

[12] Vijaykumar R., Saboo A. (2014). Review of different methods used for confirmation of endotracheal tube placement in newborns. J. Neonatal Biol..

[13] Sethi A.K., Salhotra R., Chandra M., Mohta M., Bhatt S., Kayina C.A. (2019). Confirmation of placement of endotracheal tube—A comparative observational pilot study of three ultrasound methods. J. Anaesthesiol. Clin. Pharmacol..

[14] Nagarajappa A., Kaur M., Samanta A., Tyagi A. (2019). Endotracheal tube fixation: Still a dilemma. J. Anaesthesiol. Clin. Pharmacol..

[15] Carlson J., Mayrose J., Krause R., Jehle D. (2007). Extubation force: Tape versus endotracheal tube holders. Ann. Emerg. Med..

[16] Gardner A., Hughes D., Cook R., Henson R., Osborne S., Gardner G. (2005). Best practice in stabilisation of oral endotracheal tubes: A systematic review. Aust. Crit. Care.

[17] Kim Y.S., Park S.H., Ryu S.J., Kim K.H., Jang T.H., Kim S.H. (1997). Displacement of the Endotracheal Tube is not Related to Its Fixation or Unflxation When the Neck is Extended or Flexed. Korean J. Anesthesiol..

[18] Divatia J., Bhowmick K. (2005). Complications of endotracheal intubation and other airway management procedures. Indian J. Anaesth..

[19] Tailleur R., Bathory I., Dolci M., Frascarolo P., Kern C., Schoettker P. (2016). Endotracheal tube displacement during head and neck movements. Observational clinical trial. J. Clin. Anesth..

[20] Tailleur R., Bathory I., Dolci M., Kern C., Schoettker P. (2014). Endotracheal tube displacement during head mobilization: 19AP4-6. Eur. J. Anaesthesiol. EJA.

[21] Kanburoglu M.K., Cizmeci M.N., Akelma A.Z., Ark N., Tatli M.M. (2014). Esophageal dislodgement of an endotracheal tube during nasopharyngeal oxygenation in a neonate with Pierre-Robin sequence: A case report. Arch. Argent Pediatr..

[22] Weiss M., Knirsch W., Kretschmar O., Dullenkopf A., Tomaske M., Balmer C., Stutz K., Gerber A., Berger F. (2006). Tracheal tube-tip displacement in children during head-neck movement—A radiological assessment. BJA Br. J. Anaesth..

[23] Bellini C., Turolla G., De Angelis L.C., Calevo M.G., Ramenghi L.A. (2019). Development of a novel reference nomogram for endotracheal intubation in neonatal emergency transport setting. Acta Paediatr..

[24] Akhgar A., Bahrami S., Mohammadinejad P., Khazaeipour Z., Hossein H. (2019). A New Formula for Confirmation of Proper Endotracheal Tube Placement with Ultrasonography. Adv. J. Emerg. Med..

[25] Chao C.-M., Sung M.-I., Cheng K.-C., Lai C.-C., Chan K.-S., Cheng A.-C., Hsing S.-C., Chen C.-M. (2017). Prognostic factors and outcomes of unplanned extubation. Sci. Rep..

[26] Lin P.-H., Chen C.-F., Chiu H.-W., Tai H.-P., Lee D.L., Lai R.-S. (2019). Outcomes of unplanned extubation in ordinary ward are similar to those in intensive care unit: A STROBE-compliant case–control study. Medicine.

[27] Jacques S.P.S. OMLC Optics Software. https://omlc.org/index.html.

[28] Yu B., Shah A., Wang B., Rajaram N., Wang Q., Ramanujam N., Palmer G.M., Dewhirst M.W. (2014). Measuring tumor cycling hypoxia and angiogenesis using a side-firing fiber optic probe. J. Biophotonics.

